# Neocortical and Hippocampal Theta Oscillations Track Audiovisual Integration and Replay of Speech Memories

**DOI:** 10.1523/JNEUROSCI.1797-24.2025

**Published:** 2025-05-21

**Authors:** Emmanuel Biau, Danying Wang, Hyojin Park, Ole Jensen, Simon Hanslmayr

**Affiliations:** ^1^Department of Psychology, University of Liverpool, Liverpool L69 7ZA, United Kingdom; ^2^School of Psychology, University of Birmingham, Birmingham B15 2TT, United Kingdom; ^3^Neuroscience, Physiology and Pharmacology, University College London, London WC1E 6BT, United Kingdom; ^4^Centre for Human Brain Health, University of Birmingham, Birmingham B15 2TT, United Kingdom; ^5^Department of Psychiatry, University of Oxford, Oxford OX2 6GG, United Kingdom; ^6^ Centre for Neurotechnology, School of Psychology and Neuroscience, University of Glasgow, Glasgow G12 8QB, United Kingdom

**Keywords:** audiovisual speech, magnetoencephalography (MEG), memory, theta oscillations

## Abstract

“Are you talkin’ to me?!” If you ever watched the masterpiece “Taxi Driver” directed by Martin Scorsese, you certainly recall the monologue during which Travis Bickle rehearses an imaginary confrontation in front of a mirror. While remembering this scene, you recollect a myriad of speech features across visual and auditory senses with a smooth sensation of unified memory. The aim of this study was to investigate how the fine-grained synchrony between coinciding visual and auditory features impacts brain oscillations when forming multisensory speech memories. We developed a memory task presenting participants with short synchronous or asynchronous movie clips focused on the face of speakers in real interviews, all the while undergoing magnetoencephalography recording. In the synchronous condition, the natural alignment between visual and auditory onsets was kept intact. In the asynchronous condition, auditory onsets were delayed to present lip movements and speech sounds in antiphase specifically with respect to the theta oscillation synchronizing them in the original movie. Our results first showed that theta oscillations in the neocortex and hippocampus were modulated by the level of synchrony between lip movements and syllables during audiovisual speech perception. Second, theta asynchrony between the lip movements and auditory envelope during audiovisual speech perception reduced the accuracy of subsequent theta oscillation reinstatement during memory recollection. We conclude that neural theta oscillations play a pivotal role in both audiovisual integration and memory replay of speech.

## Significance Statement

This study tests our phase-modulated plasticity hypothesis, proposing that fine-grained synchrony between lip movements and speech sounds within theta oscillations determines the formation of multisensory speech memories. We assumed that theta synchrony increases chances for the two visual and auditory inputs to arrive at the same theta phase in the hippocampus and to be associated in memory. We found that audiovisual asynchrony impacted neocortical and hippocampal theta oscillations during movie encoding. Furthermore, desynchronizing lip movements and speech sounds relative to the dominant theta oscillation in the movies disrupted subsequent theta reinstatement during memory recall. These findings demonstrate the critical role of neural theta oscillations in audiovisual speech integration and memory replay, offering insights into how we memorize multisensory experiences.

## Introduction

The role of audiovisual synchrony has been extensively investigated in perception. One definition of “synchrony” is that two events are synchronous if they happen at the same time, whereas asynchrony means that they do not occur simultaneously. Previous studies showed that the synchrony between visual and auditory inputs on theta rhythms during encoding predicted successful audiovisual associations in memory (Clouter et al., 2017; Wang et al., 2018). In the brain, similar theta rhythms originate from neuron assemblies during perception and are thought to coordinate neurons’ firing in the memory regions (Fries et al., 2001; Buzsáki, 2006, 2010, 2013; Hanslmayr et al., 2019). In the medial–temporal lobe, the hippocampus receives inputs from multiple sensory neocortices (Mayes et al., 2007; Moscovitch, 2008), and theta oscillations are thought to regulate two fundamental synaptic plasticity mechanisms supporting multisensory associations in episodic memory: In essence, the theta phase at which sensory inputs from the neocortex reach the ongoing theta oscillations in the hippocampus will determine long-term potentiation (LTP) or depression (LTD; Huerta and Lisman, 1995; Hölscher et al., 1997). Furthermore, the relative coincident spiking occurring within short time intervals across multiple inputs leads to LTP/LTD via spike-timing–dependent plasticity (Bi and Poo, 1998; Fell and Axmacher, 2011; Parish et al., 2018; Wang et al., 2023). Based on this and applying the same definition of synchrony as above, we hypothesized that the role of audiovisual synchrony upon theta rhythms [4–8 Hz] extends to speech memories as well. First, the regular syllable onsets impose comparable rhythms in continuous speech that oscillates at a preferred theta rate (Giraud and Poeppel, 2012). Previous EEG studies showed that the primary auditory cortex tracks and synchronizes with speech envelope’s theta phase during perception (Peelle and Davis, 2012; Gross et al., 2013; Park et al., 2015; Etard and Reichenbach, 2019). Second, the visual speaker’s lip movements carry equivalent syllable information at theta rate and align with sound fluctuations in multisensory speech (Ding et al., 2016; Park et al., 2016; Bourguignon et al., 2020; Biau et al., 2021). Consequently, lip openings and envelope amplitude increases naturally occur simultaneously in the synchronous condition but not in the asynchronous condition. Unsurprisingly, the coherence between the phase of lip movements and auditory envelope during spontaneous audiovisual speech is maximal at ∼6 Hz, which corresponds to the theta syllable rate (Chandrasekaran et al., 2009; Park et al., 2016). Similarly, the visual cortex tracks and synchronizes with theta oscillations carried by the speaker’s lips during speech perception (Park et al., 2016; Biau et al., 2021). Therefore, listeners contemporarily process two information streams synchronizing upon theta oscillations during audiovisual speech perception. Within this “phase-modulated plasticity” framework, we assumed that if the two visual and auditory inputs arrive at the same time in the hippocampus, then their chances of being associated are higher because they will fall into the same theta phase which favors memory formation, which is not the case for asynchronous speech. We successfully applied this assumption in previous studies using unrelated pairs of movie and sound stimuli presented in theta synchrony or asynchrony (Clouter et al., 2017; Wang et al., 2018). Here, we aim to extend this approach to speech stimuli. This begs the question of whether the brain uses endogenous theta oscillations to form integrated memories of audiovisual speech episodes. To address this question, we specifically manipulated the temporal alignment between visual and auditory streams with respect to the dominant theta oscillation in movie clips. We predicted that (1) the specific asynchrony of the theta frequency aligning audiovisual information in the movies should decrease subsequent speech memory performances. (2) Regions that integrate audiovisual information in the neocortex and hippocampus should track audiovisual synchrony with higher theta power for synchronous than asynchronous movies. (3) The same theta patterns encoded during the movies are reinstated during memory retrieval, with their fidelity depending on audiovisual synchrony during speech encoding.

## Materials and Methods

### Participants

Thirty healthy participants took part in the experiment (mean age, 24.17 years ± 2.71; nine females). All participants were English native speakers and right-handed. All of them reported normal or corrected-to-normal vision and hearing. All participants received financial reimbursement for taking part in the experiment. Three participants were excluded because of technical issues affecting the presentation of sounds during the experiment; four additional participants were excluded because of excessive noise in the data. This left 23 datasets for analysis. All participants gave written informed consent. Ethical approval was granted by the University of Birmingham Research Ethics Committee complying with the Declaration of Helsinki.

### Audiovisual speech signal analysis

Eighty-eight 5 s audiovisual movies were extracted from natural face-to-face interviews downloaded from YouTube (www.youtube.com). Satisfying movies containing meaningful content (i.e., at least one complete sentence, speaker facing toward the camera) were edited using Shotcut (Meltytech). For each movie, the video and the sound were exported separately (video, .mp4 format, 1,280 × 720 resolution, 30 fps, 200 ms linear ramp fade in/out; audio, .wav format, 44,100 Hz sampling rate, mono).

#### Lip movement and auditory speech envelope analysis

**Figure 1.**
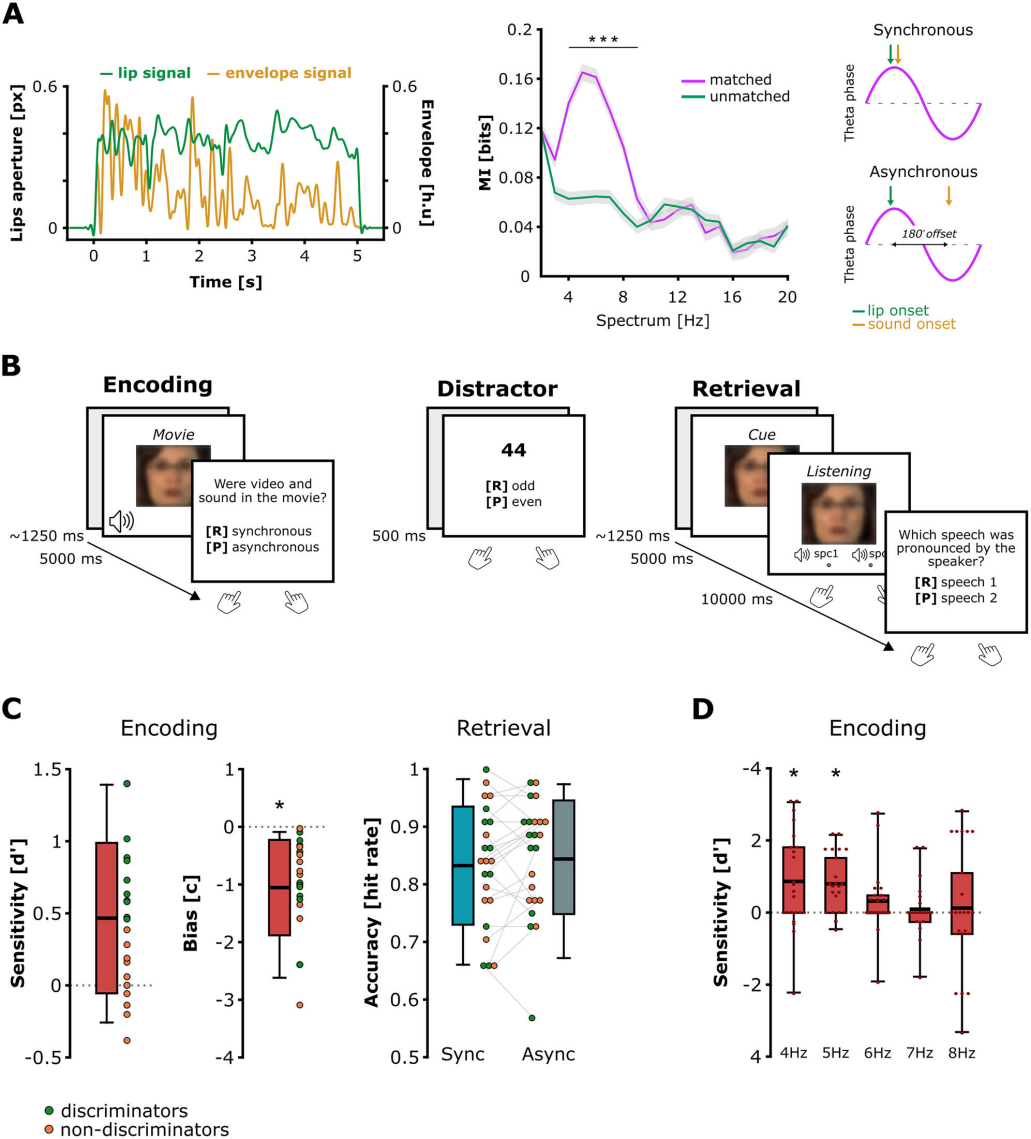
Movie stimuli, experimental design, and behavioral performances. ***A***, Left, Example of normalized lip movement (green line; pixel units) and auditory speech envelope (orange line; Hilbert units) time-series from the same movie. Center, Coupling between the lip and auditory envelope signals in the movies (MI). The lip movements and speech envelopes predominantly synchronized on 4–8 Hz theta rhythms in the movies (purple line, mean ± standard error mean). This theta coupling disappeared when lip and speech envelope signals came from different movies (green line, mean ± standard error mean). Right, Illustration of the absolute theta timing manipulation induced between lip movement and auditory envelope onsets in order to create the synchronous and asynchronous conditions. ***B***, Example of one block of the procedure consisting in a sequence composed of encoding (speech movie presentations), distractor (rehearsal prevention), and retrieval (memory test on the movies perceived during the encoding). Participants saw a clear image of the face, which is blurred here for anonymity purpose. ***C***, Behavioral performance during encoding and retrieval. The sensitivity to theta audiovisual synchrony during movie encoding was assessed with the *d*′, and the bias to respond “synchronous” independently from the actual synchrony in the movies was assessed with the *c* criterion. Memory accuracy was assessed using the hit rate (i.e., where participants selected the correct speech stimulus pronounced by the speaker depicted during the cueing; 1 = 100% correct). Participants with a sensitivity greater than the *d*′ median (0.461) during the encoding were labeled as discriminators (green dots), while participants with a sensitivity lower than the median were labeled as nondiscriminators (orange dots). ***D***, Sensitivity to audiovisual synchrony depending on the dominant frequency aligning lip movements and speech envelope in the movies during encoding. Participants were only sensitive to audiovisual synchrony in movies for which the lip movements and speech envelope aligned on slower theta frequencies (i.e., 4 and 5 Hz). Nevertheless, the memory performances were not later influenced by the dominant theta frequency at which lip and sounds were desynchronized in the movies, even when participants were sensitive to the theta timing in the lower 4–5 Hz frequencies (memory performances split by dominant frequency are not plotted). The boxplots represent the mean ± standard deviation and the error bars indicate the 5th and 95th percentiles. The dots represent individual means. Significant contrasts are highlighted with asterisks (*p < 0.05; ***p < 0.001).

Lips contour and amplitude envelope signals were extracted for each movie using in-house MATLAB codes (Fig. 1*A*, **left**). For the lip movements, we computed and used the vertical aperture information of the lips contour (i.e., aperture between the superior and inferior lips; Park et al., 2016). For the corresponding amplitude envelope, eight equidistant frequency bands spanning on the cochlear map in the range 100–10,000 Hz were constructed (Smith et al., 2002). Narrowband sound signals were bandpass filtered with a fourth-order Butterworth filter (forward and reverse). Absolute Hilbert transform was applied to obtain amplitude envelopes for each narrowband, which were averaged across bands and resulted in a unique wideband amplitude envelope per sound signal. Finally, the lip movement and auditory envelope time-series were resampled at 250 Hz for further analyses.

#### Mutual information between lip movements and auditory speech envelope

We identified the dominant activity best aligning lip movements and auditory envelope in each individual movie by applying mutual information (MI) approach (Shannon, 1948). MI quantifies phase dependence between the two speech signals for each frequency of the spectrum by using the multivariate Gaussian Copula Mutual Information (GCMI) estimator [see Ince et al. (2017) for further details]. We applied GCMI analysis to select and validate our movie clips, for which lip and sound information best aligned in the theta [4–8 Hz] range. First, we computed MI between corresponding lip and auditory envelope signals from the same audiovisual speech clip (matched). Second, we performed a peak detection of the MI within the spectrum [1–20 Hz] to determine the preferred frequency best aligning lip movements and auditory envelope in each individual movie (mean frequency MI peak across stimuli, 5.750 ± 1.341 Hz; 20 videos with a peak of MI at 4 Hz; 22 videos with a peak of MI at 5 Hz; 16 videos with a peak of MI at 6 Hz; 20 videos with a peak of MI at 7 Hz; 10 videos with a peak of MI at 8 Hz). Third, we computed MI between the lip movement signal of each stimulus and a randomly paired auditory envelope signal extracted from a different stimulus (unmatched). One-tailed *t* test comparisons of the averaged MI across the 1–20 Hz spectrum between matched and nonmatched signals confirmed a significant difference localized in the 4–9 Hz theta band (4 Hz, *t*_(19)_ = 5.846; *p* = 8.580 × 10–7; 5 Hz: *t*_(19)_ = 6.561; *p* = 3.672 × 10–8; 6 Hz, *t*_(19)_ = 5.724; *p* = 1.452 × 10–6; 7 Hz, *t*_(19)_ = 4.810; *p* = 6.272 × 10–5; 8 Hz, *t*_(19)_ = 5.064; *p* = 2.270 × 10–5; 9 Hz, *t*_(19)_ = 2.976; *p* = 0.038). For the remaining frequencies of the spectrum (i.e., from 1 to 3 Hz and from 10 to 20 Hz), the MI was not different between matched and nonmatched signals. All the *p* values were Bonferroni-corrected for multiple comparisons (Fig. 1*A*, **center**). These results confirmed that lip movement and auditory envelope signals aligned preferentially in the theta band as compared with the rest of the spectrum (Chandrasekaran et al., 2009; Park et al., 2016), which validates the selected audiovisual speech stimuli. In our study, we only selected movies with MI peaking within the theta range [4–8 Hz].

### Audiovisual theta synchrony and asynchrony in the movie stimuli

We created two versions of the 88 video clips (Fig. 1*A*, **right**): In the synchronous version, the natural synchrony between video and sound signals from the original clips was intact. Lip movements and speech envelope fluctuations aligned on their preferred theta oscillation imposed by syllable onsets. To create the asynchronous version, we shifted the onset of the auditory signal with respect to the visual signal by 180° of the preferred theta oscillation. This delay in absolute time depended on the preferred theta oscillation aligning lip movements and the auditory envelope signals determined by MI analysis. For instance, if lip movements and the speech envelope were best aligned at 4 Hz in the Movie *n*, the onset of the auditory signal was delayed by 125 ms with respect to its original timing to induce an offset with the visual signal onset corresponding to 180° in the theta phase. Instead, if lip movements and the speech envelope were best aligned at 5 Hz in another Movie *n* *+* 1, the auditory signal onset was delayed by 100 ms to create the asynchronous version of this Movie *n* *+* 1. The exact same logic was applied to create the asynchronous version of other audiovisual clips in which lip movement and auditory envelope signals aligned best at 6, 7, or 8 Hz, by inducing a delay of, respectively, 83, 71, or 63 ms. Finally, the natural order of signal presentation was always kept across stimuli and conditions, i.e., lip aperture leading its corresponding sound. Previous studies showed that subsequent memory performance decreased for stimuli encoded in audiovisual asynchrony, independently from the offset between visual and auditory onsets, i.e., 90, 180 or 270° (Clouter et al., 2017; Wang et al., 2018). To limit the number of movie versions and straightforward comparison here, we chose to create a unique asynchronous condition with a 180° offset.

### Experimental design

#### Audiovisual speech memory task

Participants seated comfortably in the magnetoencephalography (MEG) gantry, ∼150 cm away from the projection screen. The task was presented on a middle-gray screen (1,920 × 1,200 pixel resolution) using Psychophysics Toolbox-3 (Brainard and Vision, 1997). The sound was presented between 70 and 80 dB through MEG-compatible insert earphones (Etymotic Research). The experimental paradigm consisted of repeated blocks, with each block being composed of three successive tasks: encoding, distractor, and retrieval tasks (Fig. 1*B*). During the encoding, participants were presented with a series of audiovisual speech movies and performed an audiovisual synchrony detection. Each trial started with a brief fixation cross (jittered duration, 1,000–1,500 ms) followed by the presentation of a random synchronous or asynchronous audiovisual speech movie (5 s). After the movie end, participants had to determine whether video and sound were presented in synchrony or asynchrony in the movie, by pressing the index finger (“synchronous”) or the middle finger (“asynchronous”) button of the response device as fast and accurate as possible. The next trial started after the participant’s response. After the encoding, the participants did a short distractor task. Each trial started with a brief fixation cross (jittered duration, 1,000–1,500 ms) followed by the presentation of a random number (from 1 to 99) displayed at the center of the screen. Participants were instructed to determine as fast and accurate as possible whether this number was odd or even by pressing the index (“odd”) or the middle finger (“even”) button of the response device. Each distractor task contained 20 trials. The purpose of the distractor task was only to clear memory up. After the distractor task, the participants performed the retrieval task to assess their memory. Each trial started with a brief fixation cross (jittered duration, 1,000–1,500 ms) followed by the presentation of a static frame depicting the face of a speaker from a movie attended in the previous encoding. During this visual cueing (5 s), participants were instructed to recall as accurately as possible every auditory information previously associated with the speaker’s speech during the movie presentation. At the end of the visual cueing, participants were provided the possibility to listen two auditory speech stimuli: one stimulus corresponded to the speaker’s auditory speech from the same movie (i.e., matching). The other auditory stimulus was taken from another random movie with the same speaker gender (i.e., unmatching). Participants chose to listen each stimulus sequentially by pressing the index finger (“Speech 1”) or the middle finger (“Speech 2”) button of the response device. The order of displaying was free, but for every trial, participants were allowed to listen to each auditory stimulus only one time to avoid speech restudy. At the end of the second auditory stimulus, participants were instructed to determine as fast and accurate as possible which auditory speech stimulus corresponded to the speaker’s face frame, by pressing the index finger (“Speech 1”) or the middle finger (“Speech 2”) button of the response device. The next retrieval trial started after the participant’s response. After the last trial of the retrieval, participants took a short break, before starting a new block (encoding–distractor–retrieval).

#### Staircase procedure and speech condition counterbalancing

In total, the paradigm presented 88 audiovisual speech stimuli with a staircase procedure adapting online to individual performances in order to limit any ceiling effect in the memory test: the encoding task of the first block always contained 16 trials. The length of the encoding task in the next block depended on the accuracy in the previous retrieval task (i.e., the percentage of auditory speech stimuli correctly retrieved with the visual cue) and was determined as follows: when accuracy in the retrieval *n* was between 70 and 80%, the encoding *n* *+* 1 remained the same. If memory accuracy in the retrieval *n* was above 80%, the length of the encoding *n* *+* 1 was increased with four more trials to increase difficulty in retrieval *n* *+* 1. If accuracy in the retrieval *n* was below 70%, the length of the encoding *n* *+* 1 was decreased with two less trials to decrease difficulty in retrieval *n* *+* 1. During the encoding, half of the total audiovisual stimuli were presented in the synchronous condition (44 trials), and the other half was presented in the asynchronous condition (44 trials). To control for material effects, i.e., some stimuli might be more memorable than others, we counterbalanced the assignment of stimuli to the synchronous or asynchronous condition across participants (e.g., the synchronous version of the Movie *n* was presented to Participant *X* and the asynchronous one to Participant *X* *+* 1).

### MEG recording and preprocessing

#### MEG acquisition

MEG data were recorded using a 306-sensor TRIUX MEGIN system with 102 magnetometers and 204 orthogonal gradiometers in a magnetically shielded room. The data were bandpass filtered online using anti-aliasing filters from 0.1 to 330 Hz and sampled at 1,000 Hz. The location of the three fiducial points (i.e., nasion, left and right preauricular points), as well as four head position indicator coils (HPI, behind left and right ears right below hairline and another two on the forehead with a respective distance of at least 3 cm distance), was digitized using a Polhemus FASTRAK electromagnetic digitizer system (Polhemus). Additionally, ∼200 extra points on each participant’s scalp were also digitally sampled to spatially coregister the off-line MEG analysis with individual structural MRI image or templates. MEG data were acquired with participants in a sitting position (MEG gantry at a 60° angle).

#### MRI acquisition

Structural MRI images (T1 weighted) were acquired for 17 participants using a 3 Tesla Siemens Magnetom Prisma scanner (MP-RAGE, TR, 2,000 ms; TE, 2.01 ms; TI, 880 ms; flip angle, 8°; FOV, 256 × 256 × 208 mm; 1 mm isotropic voxel). For the remaining six participants without individual T1-weighted structural MRI image, we used the MNI template brain from FieldTrip.

#### MEG preprocessing and head movement detection

Data preprocessing and analyses were performed in MATLAB R2018 (MathWorks), by using the FieldTrip toolbox (Oostenveld et al., 2011) and custom-made scripts. Only gradiometer data were included in the analysis. Data were first bandpass filtered at 0.1–165 Hz to exclude the signal generated by the HPI coils. Second, the continuous data were epoched at the events of interest (encoding, movie onset; retrieval, visual cue onset, first and second auditory speech onsets; localizer tasks, silent movie and auditory speech onsets), with each epoch beginning 2,000 ms before the stimulus onset and ending 2,000 ms after the stimulus onset (total epoch duration, 9,000 ms). The duration of the epochs was longer than the windows of interest to exclude potential filter artifacts but was subsequently restricted to windows of interest in the center of the epochs when conducting statistical analysis. Third, eyeblink or cardiac components were identified by applying independent components analysis and removed. Finally, data were visually inspected, and any artifactual epochs or sensors were removed from the dataset (mean percentage of trials removed, 10%; range, 1–25%; mean number of malfunctioning sensors removed, 1.43; range, 0–6). Participants with extreme head motion during MEG recordings were identified as follows: (1) Participants’ data were high-pass filtered to 250 Hz to isolate the cHPI signal. (2) For each sensor, the variance of the signal was calculated across time-points of the continuous data. (3) The mean variance was averaged across sensors to provide the individual estimate of change in cHPI signal. (4) The mean variance and its standard deviation were calculated across participants. (5) Datasets with a variance greater than three standard deviations above the group mean were excluded from analysis.

#### Data reconstruction in the source space

Preprocessed data were reconstructed in the source space using individual head models and structural MRI (T1-weighted) scans for 17 participants. For the six participants without structural MRI scans, we used a standard head model and MRI scan templates from the FieldTrip toolbox. Data were reconstructed using a single-shell forward model and a linearly constrained minimum variance (LCMV) beamformer (van Veen et al., 1997). The lambda regularization parameter was set to 1%. The source model covered the whole brain with virtual electrodes spaced 10 mm apart in all planes.

#### Time–frequency decomposition in the source space

Time–frequency decomposition was performed at the source level on the encoding and cueing epochs as follows: (1) The preprocessed data were convolved with a six-cycle wavelet (from −1 to +5 s with respect to the stimulus onset, in steps of 50 ms; 1–40 Hz; in steps of 1 Hz). (2) The background 1/*f* characteristic was subtracted using an iterative linear fitting procedure to isolate oscillatory contributions (Manning et al., 2009; Zhang and Jacobs, 2015; Griffiths et al., 2021). A vector containing values of each derived frequency *A* and another vector containing the power spectrum averaged over all time-points and trials of the encoding *B* were log-transformed to approximate a linear function. The linear equation *Ax* = *B* was solved with least-square regression, in which *x* is an unknown constant describing the 1/*f* characteristic. The 1/*f* fit (*Ax*) was then subtracted from the log-transformed power spectrum (*B*). An iterative algorithm removed peaks in this 1/*f*-subtracted power spectrum that exceeded a threshold determined by the mean value of all frequencies sitting below the linear fit. As the power spectrum is the summation of the 1/*f* characteristic and oscillatory activity, every value below the linear fit can be seen an estimate error of the fit. Any peaks exceeding this threshold were removed from the general linear model, and the fitting was repeated. This step was applied to avoid fit bias due to outlying peaks (Donoghue et al., 2020).

### Statistical analysis

#### Behavioral performance and statistical analysis

(1) Audiovisual synchrony detection: trials for which participants correctly detected audiovisual synchrony between video and sound in the synchronous condition were labeled as “hit” (i.e., participants responded “synchronous” in the synchronous audiovisual speech movies). Trials for which participants wrongly responded synchronous in the asynchronous condition were labeled as “false alarm.” Participants’ individual sensitivity to audiovisual theta timing during movie encoding was assessed by computing the *d*′ from the audiovisual synchrony detection task. (2) Memory retrieval: trials for which participants correctly recalled the auditory speech stimulus associated with the visual cue were labeled as “hit.” In contrast, trials for which participants did not recall the correct auditory stimulus were labeled as “miss.” Subsequent memory accuracy and reaction times were computed for each participant by averaging hit rates and their associated reaction times during the retrieval.

#### MEG statistical analysis

*Whole-brain difference of theta power between synchronous and asynchronous encoding*. We limited the analysis to a time-window of +1 to +4 s with respect to the movie onset to avoid biases induced by the onset and offset of the movie presentation. For every trial, the power spectrum was centered on the frequency corresponding to the peak of MI between lip and auditory envelope signals determined in the movie analyses (±3 Hz). This step was done to be able to average all the trials together considering the main theta activity carried in each individual movie. For instance, if lip and envelope aligned best at 4 Hz in a Movie *n*, the realigned spectrum of the MEG epoch corresponding to the encoding of Movie *n* was now 4 ± 3 Hz (from 1 to 7 Hz; 1 Hz bin), ensuring that the central bin of each single trial corresponds to the objectively determined frequency peak of theta activity. Then, the realigned theta power spectrum was averaged across trials within every participant for group analyses. For each participant, we calculated the difference of theta power between the synchronous minus asynchronous condition (theta_sync_ − theta_async_) in the central bin of the realigned spectrum at all virtual sensors. The difference of theta power at the group level was statistically assessed against zero by performing a one-tailed cluster–based permutation test (2,000 permutations; α threshold = 0.05; cluster α threshold = 0.05; minimum neighborhood size, 3). Cohen’s *d*_z_ was used as the measure of the effect size for significant clusters only as follows: *d*_z_ = mean *t* statistic within the cluster divided by the square root of the number of participants (Lakens, 2013). The anatomical regions with maximum voxel activation were defined using the automated anatomical labeling atlas (Tzourio-Mazoyer et al., 2002).

*Difference of theta power in the hippocampus between synchronous and asynchronous encoding*. For every participant, the mean difference of theta power theta_sync_ − theta_async_ was averaged across the hippocampal sources. Hippocampal virtual sensors (left + right) were defined using the *wfupickatlas* toolbox for SPM (hippocampal region of interest, 32 virtual sensors). The mean difference of theta power across participants was statistically assessed against the null hypothesis (*t* = 0) by applying a one-sample *t* test (one-tailed). To further address whether the difference of theta power was specific to the dominant frequency aligning lip movements and auditory envelope in the stimuli, we compared the original hippocampal difference of theta power against the hippocampal differences in power of neighboring frequencies as follows: (1) The power was extracted in the frequencies corresponding to the ones determined with the MI analyses, divided or multiplied by two times the golden mean 1.618 to generate low (4/3.24 = 1.24 Hz; 5/3.24 = 1.55 Hz; 6/3.24 = 1.85 Hz; 7/3.24 = 2.16 Hz; 8/3.24 = 2.47 Hz) and high (4 × 3.24 = 12.94 Hz; 5 × 3.24 = 16.18 Hz; 6 × 3.24 = 19.42 Hz; 7 × 3.24 = 22.65 Hz; 8 × 3.24 = 25.88 Hz) neighboring frequencies. Dividing or multiplying by two times the golden mean ensured that the low-high neighbor frequencies did not share any harmonic with the original frequencies (Pletzer et al., 2010). (2) As for the original data, the power spectrum for each trial was recentered on the corresponding low or high frequency to create two temporary data, i.e., power in the low- or high-frequency band. The realigned power was averaged across trials in the synchronous and asynchronous conditions separately, across hippocampal virtual sensors in the low [1.24–2.47 Hz] and high [12.94–25.88 Hz] bands. The difference of power between synchronous minus asynchronous conditions was computed and averaged across low and high neighboring frequencies for every participant. Finally, the difference of power difference between the original theta and the neighboring frequencies was statistically assessed by means of a one-sample *t* test (one-tailed).

*Theta phase coupling between the neocortex and hippocampus*. We used theta phase coupling to test whether lip-sound synchrony in the movies impacted the synchronization between the hippocampus and the sensory regions of the neocortex (auditory and visual cortices) during movie encoding. We included one auditory source, one visual source, one hippocampal source, and one source from the cerebellum as a control region in the phase coupling analyses. We selected the cerebellum as the null region, as we did not have any specific directional hypothesis on how audiovisual speech perception modulates the neural theta oscillations in this region or its connectivity with the hippocampus. First, for each participant, we individually selected the preferred auditory source among the virtual sensors contained in the left auditory cortex (total virtual sensors, 12) exhibiting the maximal theta phase similarity between the MEG signal and the auditory envelop, reflecting auditory tracking during synchronous movie encoding (from +1 to +4 s with respect to the movie onset). Similarly, the preferred visual source was selected among the virtual sensors contained in the left visual cortex (total virtual sensors, 70) as the one exhibiting the maximal theta phase similarity between the MEG signal and the lip movements, reflecting visual tracking during synchronous movie encoding. The preferred hippocampal source was determined as the source exhibiting the maximal theta power in the hippocampus (total virtual sensors, 16) during movie encoding. The null-region source was selected within the left cerebellum (total virtual sensors, 1). Second, the signals from left auditory, visual, and cerebellum sources were projected orthogonally onto the hippocampal signal applying a Gram–Schmidt process for single trials before computing phase information. This was done to reduce the noise correlation patterns reflecting activity from a common source estimate captured at different electrodes. Third, for each trial, the instantaneous theta phase of the hippocampal and orthogonalized time-series (auditory, visual, and cerebellum) were computed by applying a Hilbert transform with a bandpass filter centered on the dominant frequency bin ±2 Hz determined individually for every movie. Fourth, the absolute difference of unwrapped instantaneous phase between the hippocampus source and the other source (auditory, visual, or cerebellum) was computed for each single trial at each time-point in the central time-window (from +1 to +4 s with respect to the movie onset). The distance between the absolute phase difference observed in the data (ϕ) and the theoretical angle *ϕ*_theo_ = 0 indexing perfect phase synchrony between two oscillations was then computed at each time-point of the trial to provide one value (*ϕ* − *ϕ*_theo_) per time-point. Sixth, the resultant vector length *r* of each trial was computed by collapsing the distance values across time-points. The vector length *r* estimates how the lag between the theta oscillations from two regions was stable during encoding, which reflects the strength of cross-region phase coupling. Steps four, five, and six were performed separately for the three cross-region interactions of interest, hippocampus–auditory cortex, hippocampus–visual cortex, and hippocampus–cerebellum, as well as for synchronous and asynchronous conditions. The modulations of cross-region phase coupling (estimated with the vector length *r*) were statistically assessed with a two-way repeated–measure ANOVA including factors regions and synchrony.

*Theta phase similarity calculation*. We calculated the phase similarity between neural theta oscillations in the auditory cortex and in the auditory envelope signal from the movie cued during the retrieval, to establish that the brain activity replays oscillatory patterns when recollecting speech memories. We applied a sliding window approach that allows to detect dynamic memory replay with different onset times, as it was not possible to determine exactly when participants retrieved speech information during successful trials (Michelmann et al., 2016). This method quantifies the phase similarity for a frequency of interest between the pairs of neural MEG signal and auditory envelope time-series with a sliding window as follows: (1) For each trial, the phase values of every time-point from the two time-series were extracted by multiplying the Fourier-transformed data with a complex Morlet wavelet of six cycles and downsampled to 64 Hz (from −3 to +7 s with respect to the onset). Crucially, the theta frequency of interest for each trial corresponded to the frequency aligning best lip movements and auditory envelope in the corresponding stimulus. (2) For the first time-point of the MEG time-series (*t*1_me*g*_), the theta phase similarity was computed between the 1 s window centered on *t*1_meg_ (±500  ms) and a fixed 1 s window centered on the first time-point of the auditory speech envelope time-series corresponding to the same speaker (*t*1_env­_ ± 500 ms). Phase similarity between *t*1_meg_ and *t*1_env_ windows was assessed following the single-trial phase locking value approach (Lachaux et al., 2000; Mormann et al., 2000). To do so, we computed the cosine of the absolute angular distance for each time-point, and we quantified the similarity as 1 minus the circular variance of phase differences over time within the *t*1_meg/env_ window. Therefore, a unique phase similarity value between 0 and 1 was obtained for *t*1_meg_. The exact same operation (2) was repeated between the fixed *t*1_env_ window and a same-length window shifted by one time-point over the MEG time-series, corresponding to a 1 s window centered on *t*2_meg_ (±500 ms). At the end of the sliding window process, we obtained the phase similarity between all time-points of the MEG epoch (from −3 to +7 s with respect to the stimulus onset) and the first time-window *t*1_env_ of the auditory envelope signal. The exact same operation was then repeated for the remaining time-windows of the envelope signal to generate a matrix of theta phase similarity in the two dimensions (i.e., MEG and auditory envelope signals), at every virtual sensor contained in the two regions of interest (i.e., left and right auditory cortex). The virtual sensors contained in the left and right primary auditory cortices of interest were defined by using the *wfupickatlas* toolbox for SPM (left Heschl’s gyrus, 12 virtual sensors; right Heschl’s gyrus, 12 virtual sensors).

*Auditory speech envelope and lip movement neural tracking during movie perception*. We applied the exact same method to establish where cortical neural oscillations tracked theta activity carried by the auditory speech envelope during movie encoding. We computed phase similarity between the MEG signal and the corresponding envelope signal of the epoch. For every trial, the phase similarity was also computed between the MEG signal and a different auditory envelope signal randomly selected from the pool of stimuli with the same dominant theta frequency, excluding the one corresponding to the same MEG epoch. The mean phase similarity was then averaged across every time-point within the central time-window (from +1 to +4 s) in the matching and unmatching cases of the synchronous and asynchronous conditions separately. The difference of averaged phase similarity across the whole brain between the matching and unmatching signals was assessed by means of a one-tailed cluster–based permutation test for the synchronous condition first, then for the asynchronous condition (2,000 permutations, α threshold = 0.05; cluster α threshold = 0.05; averaged across virtual sensors).

*Auditory speech envelope reinstatement during retrieval*. We tested whether the replay of auditory speech was modulated by audiovisual synchrony during movie encoding as follows: (1) Theta phase similarity matrix was averaged across trials in the synchronous and asynchronous conditions separately at the virtual sources contained in the auditory cortices (left and right Heschl’s gyrus separately) for every participant. The number of trials averaged within condition was counterbalanced with a subsample corresponding to the smallest number of available trials between synchronous and asynchronous conditions (mean trial number in synchronous condition, 33.09 ± 6.10, and asynchronous condition, 31.17 ± 6.58). (2) The theta phase similarity difference between synchronous and asynchronous retrieval (from 0 s to +5 s with respect to the cueing onset) was statistically assessed at the group level by means of a cluster-based permutation test (2,000 permutations, α threshold = 0.05; cluster α threshold = 0.05; one-tailed). To source localize the theta phase similarity difference during the successful retrieval, we averaged the phase similarity across every time-point contained within a time-window centered on the maximum *t* value coordinates from the significant cluster revealed in the left auditory cortex (±500 ms in the MEG and auditory envelope signals), for the synchronous and asynchronous conditions. The difference of theta phase similarity between synchronous and asynchronous retrieval was then statistically assessed by performing a one-tailed cluster–based permutation test at the group level (2,000 permutations, α threshold = 0.05; cluster α threshold = 0.05).

Finally, we tested whether speech stimuli replay took place during retrieval, independently from the condition of speech encoding (synchronous vs asynchronous). We compared theta phase similarity during visual cueing against the chance level as follows: (1) For every participant, the original theta phase similarity was computed from synchronous and asynchronous trials together, with the number of trials from the two conditions being counterbalanced by taking the smallest number of available trials between synchronous and asynchronous. (2) The original theta phase similarity matrix was averaged across trials and across virtual sensors from the Heschl’s gyrus (left and right together). (3) One hundred permuted data were generated by applying steps (1) and (2) after mismatching the corresponding pairs of neural and stimulus signals. For every trial, the theta phase similarity was computed between the corresponding MEG signal and a random auditory envelope signal. The auditory envelope was randomly selected from the pool of stimuli with the same dominant theta frequency, excepting the one corresponding to the same MEG epoch. The permuted theta phase similarity matrix was averaged across trials and sources contained in the Heschl’s gyrus (left + right). This step was repeated 100 times to generate 100 permuted phase similarity data per participant. (4) The permuted theta phase similarity matrix was averaged across the 100 permuted phase similarity data to obtain a phase similarity value per participant under the null hypothesis. (5) The difference between the original and the permuted theta phase similarity matrices was statistically assessed (from 0 to +5 s with respect to the cueing onset) at the group level with a one-tailed cluster–based permutation test (2,000 permutations, α threshold = 0.05; cluster α threshold = 0.05).

## Results

### Audiovisual theta synchrony during speech encoding did not affect subsequent memory accuracy

We computed the performance scores of the participants during the encoding and retrieval phases to address our first prediction (Fig. 1*C*). In the memory task, participants correctly recalled the auditory speech associated with the speaker’s face during the retrieval (synchronous, mean hit rate, 0.833 ± 0.103; asynchronous, mean hit rate, 0.848 ± 0.099) and above chance level (i.e., 0.5 accuracy) in both synchronous (*t*_(22)_ = 15.551; *p* < 0.001; Cohen’s *d* = 3.243; one-tailed) and asynchronous conditions (*t*_(22)_ = 16.781; *p* < 0.001; Cohen’s *d* = 3.499; one-tailed). Nevertheless, results revealed no significant difference of accuracy between the two conditions behaviorally (*t*_(22)_ = −1.005; *p* = 0.326; Cohen’s *d* = −0.209; two-tailed). Therefore, the theta asynchrony between lip and sounds during speech encoding did not significantly impair the formation of speech memory (Fig. 1*C* **right**). Participants’ sensitivity to theta timing during movie encoding was assessed with the *d’* from the audiovisual synchrony detection task (Fig. 1*C*, **left**). The averaged *d*′ was close to zero (mean *d*′ = 0.467 ± 0.522) but significantly positive (*t*_(22)_ = 4.29; *p* < 0.001; Cohen’s *d* = 0.895; one-tailed). This result suggested that participants were able to distinguish between synchronous and asynchronous movies, although they had a bias to respond synchronous more often. A one-sample *t* test against zero confirmed that the averaged *c* criterion (mean *c* = −1.054 ± 0.827) was significantly below zero and that participants indeed tended to respond more systematically “synchronous” across trials (*t*_(22)_ = −6.112; *p* < 0.001; Cohen’s *d*_z_ = 1.802; two-tailed).

The synchrony detection performance during movie encoding suggests differences of sensitivity among participants (Fig. 1*C*). To test whether sensitivity to audiovisual alignment influenced subsequent memory, we split the participants in two populations of synchrony discriminators (green dots) and nondiscriminators (orange dots), with a *d*′, respectively, above and below the d′ median (0.461). We statistically assessed any difference of memory performance between the two groups (discriminators vs nondiscriminators) and conditions (synchronous vs asynchronous) with a two-way repeated–measure ANOVA. Results revealed no significant effect of the group (*F*_(1,19)_ = 0.043; *p* = 0.841; *η²* = 0.004), condition (*F*_(1,19)_ = 1.236; *p* = 0.292; *η²* = 0.110), or interaction group × condition (*F*_(1,19)_ = 0.013; *p* = 0.912; *η²* = 0.001) on subsequent memory performance. Therefore, the sensitivity to audiovisual theta synchrony did not predict subsequent memory performance.

Finally, participants’ sensitivity to audiovisual synchrony may depend on the frequency at which lip and sounds were desynchronized in the movies. In particular, participants might differentiate synchronous from asynchronous movies in the lower frequencies (i.e., 4–5 Hz with audiovisual offsets corresponding to respectively 125 and 100 ms), but not the faster ones. If so, differences of sensitivity to audiovisual theta synchrony during encoding might affect subsequent memory differently as well. To address this possibility, we split the synchrony detection performance during encoding (*d*′) by the dominant frequency aligning lip movements and speech envelope at either 4, 5, 6, 7, or 8 Hz. We first tested whether the dominant frequency had an effect on the *d*′ by means of a one-way ANOVA with the fixed factor frequency (4, 5, 6, 7, or 8 Hz). Results revealed no significant effect of frequency on *d*′, although a strong tendency (*F*_(4,110)_ = 2.33; *p* = 0.060; *η²* = 0.078). We therefore compared each averaged *d*′ against zero with independent one-sample *t* tests to establish whether participants’ sensitivity to the audiovisual synchrony differed accordingly (Fig. 1*D*). Results confirmed that the averaged *d*′ was only significantly greater than zero for the slower 4 and 5 Hz frequencies (mean *d*′ 4 Hz, 0.863 ± 1.352; *t*_(22)_ = 3.061; *p* = 0.029; Cohen’s *d* = 0.638; mean *d*′ 5 Hz, 0.797 ± 0.816; *t*_(22)_ = 4.682; *p* = 0.001; Cohen’s *d* = 0.976; mean *d*′ 6 Hz, 0.317 ± 0.898; *t*_(22)_ = 1.693; *p* = 0.523; Cohen’s *d* = 0.353; mean *d*′ 7 Hz, 0.093 ± 0.746; *t*_(22)_ = 0.599; *p* > 0.9; Cohen’s *d* = 0.125; mean *d*′ 8 Hz, 0.122 ± 1.683; *t*_(22)_ = 0.349; *p* > 0.9; Cohen’s *d* = 0.073; *p* values were Bonferroni-corrected for multiple comparisons). We then tested whether differences of sensitivity to audiovisual theta synchrony had an effect on subsequent memory, by means of a two-way repeated–measure ANOVA with the two-factor condition (synchronous vs asynchronous) and frequency (4, 5, 6, 7, or 8 Hz). Results revealed no significant effect of condition (*F*_(1,22)_ = 0.284; *p* = 0.599; *η²* = 0.013), frequency (*F*_(1,22)_ = 1.065; *p* = 0.379; *η²* = 0.046), or interaction between condition and frequency (*F*_(1,22)_ = 1.290; *p* = 0.280; *η²* = 0.055). These results showed that different rates of audiovisual asynchrony during movie encoding did not impact subsequent memory differently, even when participants were sensitive to the theta timing in the slower frequencies.

### Neocortical and hippocampal oscillations reflect visual lip and sound information integration via theta phase

**Figure 2.**
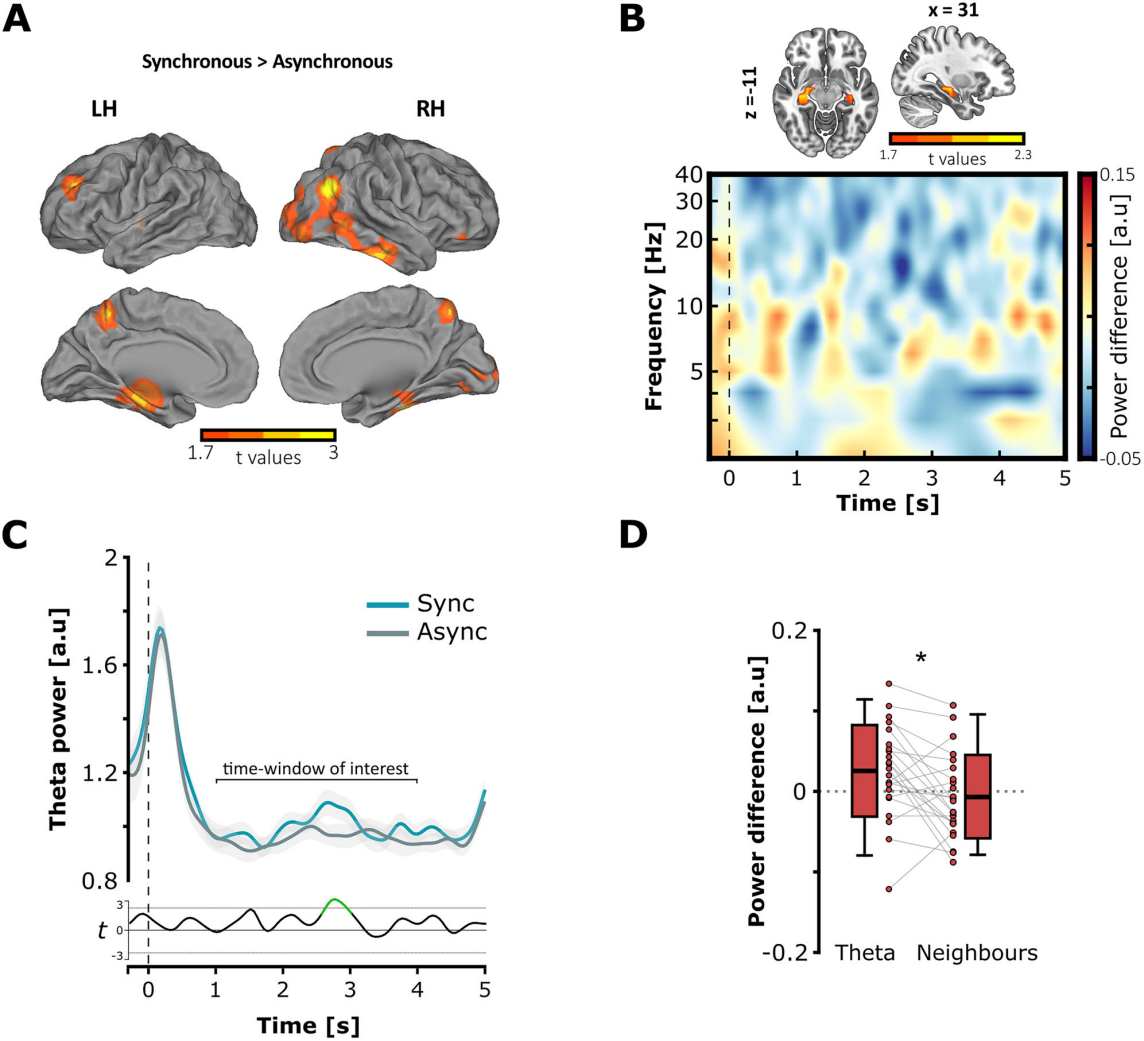
Brain oscillations are modulated in the neocortex and the hippocampus by the audiovisual theta timing during movie encoding. ***A***, Source localization of the difference of theta power in response to the audiovisual asynchrony in the movies (synchronous minus asynchronous). The perception of asynchronous movies induced a decrease of theta power as compared with synchronous movies, in cortical regions associated with language network (threshold at significant *t* values; cluster-corrected at α threshold = 0.05). ***B***, **Top**, Statistical difference of theta power in the hippocampus in response to the audiovisual asynchrony (*x* = 31; *y* = −19; *z* = −11; threshold at significant *t* values; cluster-corrected at α threshold = 0.05). **Bottom**, Logarithmic time–frequency plot of nonrealigned power spectrum difference in the hippocampus. The time–frequency only plots the spectral power difference for visualization purpose and does not report any statistical difference. ***C***, Temporal modulation of theta power in the hippocampus during the encoding of synchronous (blue line, mean ± standard error mean) and asynchronous movies (gray line, mean ± standard error mean). The bottom line (black) depicts the *t* values between the two cases for each time-point of the movie encoding (−0.3 s to 5 s with respect to the picture onset). The significant time-points are depicted in green (multiple-comparison corrected *p* values). In ***B*** and ***C***, the vertical dashed lines at zero on the *x*-axis indicate the movie onset. ***D***, Mean difference of hippocampal power in the theta range versus neighboring frequencies in the central time-window of interest. Theta oscillations in the hippocampus were specifically impacted by the audiovisual synchrony at the dominant frequencies (i.e., where lip movements and auditory envelopes were aligned in the movies). The boxplots represent the mean ± standard deviation and the errors bars indicate 5th and 95th percentiles. The dots represent individual means (*p < 0.05).

To test our second prediction, we first investigated how theta oscillations in the neocortex reflect the temporal alignment between visual and auditory speech signals on the dominant theta frequency during movie perception. We contrasted theta power responses between the synchronous and asynchronous conditions at the whole brain level. Crucially, the power spectrum in every trial was first realigned on the frequency corresponding to the peak of MI between the lip and auditory envelope signals determined in the movie analyses (Fig. 1*A*). This step was critical in order to average all the trials together considering the main theta activity carried in each individual movie before performing oscillation analyses. The cluster-based analysis revealed a significant cluster which established that the perception of asynchronous audiovisual speech induced a decrease of theta power as compared with synchronous speech (*p* = 0.047; cluster size, 668.76; mean *t* statistic within cluster, 2.164; Cohen’s *d*_z_ = 0.451; from +1 to +4 s with respect to the movie onset). The difference of theta power was localized bilaterally in the temporal and medial–temporal regions, in the right superior and inferior parietal lobules, and in the left frontal gyrus (Fig. 2*A*). These results established that the multisensory language network integrated the natural synchrony between lip movements and auditory envelope at theta oscillations. Here, we measured multisensory speech integration with theta power as it represents direct readout of the phase synchrony between the two sensory regions, which project into the associative neocortical regions.

Furthermore, we addressed whether audiovisual asynchrony in the movies impacted theta oscillations in the hippocampus as well. In line with our hypothesis, the natural temporal alignment of auditory and visual inputs on theta should also synchronize neural responses in the hippocampus where coinciding information is associated for memorization. Accordingly, a better orchestration of neural theta oscillations in the hippocampus would be reflected by a greater theta power during synchronous compared with asynchronous speech. We computed the mean difference of theta power between synchronous and asynchronous conditions, averaged within the central time-window during movie encoding (from +1 to +4 s with respect to the onset) and across the virtual sensors from the hippocampus (left + right; Fig. 2*B*, **top**). A one-tailed cluster–based permutation test revealed a significant positive cluster (*p* = 0.024; cluster size, 19.201; mean *t* statistic within cluster, 2.133; Cohen’s *d*_z_ = 0.445). This result confirmed that audiovisual asynchrony in the movies impacted theta power down to the hippocampal region as well (Fig. 2*B*, **top**). Figure 2*B*, bottom, plots the time–frequency of the spectral power difference between synchronous minus asynchronous movie encoding in the hippocampus. Figure 2*C*, **top**, depicts the temporal modulation of theta power in the hippocampus during movie presentation (from −0.3 to +5 s with respect to the movie onset), after realigning the spectrum on the peak frequency of each single trial, i.e., where lip movements and auditory envelope information showed maximal MI. Theta power time-course was averaged across successful trials and hippocampal sources in the synchronous and asynchronous conditions for each participant. A cluster-based permutation test was then performed on the theta power pooled across participants to determine any difference between synchronous and asynchronous conditions through movie encoding. Results indicated a significant positive cluster (*p* = 0.049; cluster size, 21.610; mean *t* statistic within cluster, 2.701; Cohen’s *d*_z_ = 0.563), confirming an attenuation of theta power localized in the central portion of interest during the encoding of asynchronous movies (Fig. 2*C*, **bottom line**). The strong theta power increase at the start of the trial (Fig. 2*C*, **top**) was likely induced by event-related responses at the onset of the movies. It is worth noting that spontaneous hippocampal theta activity preceded the movie onsets, which is not surprising as theta oscillations dominate in the hippocampus (Buzsáki, 2006; Griffiths et al., 2019). Most relevant to our hypothesis, we found that hippocampal theta power was decreased by the audiovisual asynchrony in the movies, as compared with synchronous condition (mean theta power difference, 0.025 ± 0.057; *t*_(22)_ = 2.11; *p* = 0.023; Cohen’s *d* = 0.44). This result confirmed that the temporal alignment between visual and auditory inputs during speech encoding also determined the level of theta synchrony between neural responses in this key region for memory. To verify whether hippocampal theta oscillations tracked specifically the dominant activity aligning visual and auditory information in the movies, we compared the magnitude of hippocampal theta power difference between the 4 and 8 Hz range of interest and neighboring frequency bands (Fig. 2*D*). To do so, we divided each original theta frequency and multiplied by two times the golden mean (1.618) to determine the same number of frequencies with no shared harmonic in the low and high neighboring bands surrounding the theta range (Pletzer et al., 2010; see Materials and Methods section). In contrast with the theta band, a one-sample *t* test showed that the difference of mean power in the neighboring frequencies was not significantly different from zero (mean power difference, −0.007 ± 0.052; *t*_(22)_ = −0.674; *p* = 0.746; Cohen’s *d* = 0.14). Furthermore, we predicted that the magnitude of mean power decrease induced by the audiovisual asynchrony should be greater in the functional theta frequencies compared with the neighboring ones. We tested our directional hypothesis by applying a paired-sample *t* test on the difference of mean power between the two bands. Results confirmed that the power difference was significantly greater in the theta 4–8 Hz frequencies of interest than in the neighboring frequencies (*t*_(22)_ = 2.866; *p* = 0.005; Cohen’s *d* = 0.587). Altogether, the results show that the fine-grained syllabic organization of audiovisual speech determined the orchestration of neural oscillations at the same theta rate during perception. Furthermore, this organization was maintained downstream in the hippocampus, which may promote the formation of audiovisual associations in memory.

**Figure 3.**
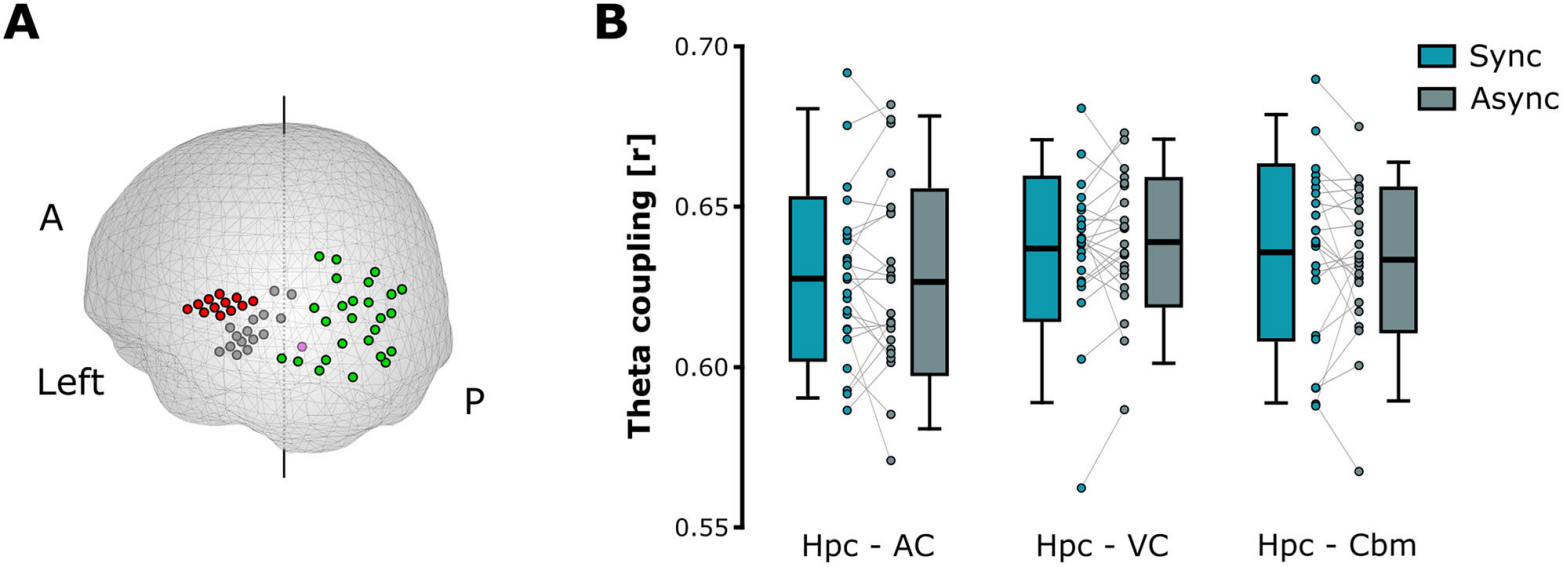
Theta phase coupling between the hippocampus and the neocortical sensory regions during movie encoding. ***A***, Visualization of the sources of interest located in the left auditory cortex (red dots), left visual cortex (green dots), left hippocampus (gray dots), and the null region (cerebellum, pink dot). Each dot corresponds to the preferred source of a single participant, but note that some preferred sources were shared across participants. ***B***, Mean theta phase coupling between the hippocampus and the three regions, respectively, left auditory cortex [hippocampus–auditory cortex (Hpc–AC)], the left visual cortex [hippocampus–visual cortex (Hpc–VC)], and the left cerebellum [hippocampus–cerebellum (Hpc–Cbm)] during the synchronous and asynchronous movies. The boxplots represent the mean ± standard deviation, and the errors bars indicate 5th and 95th percentiles. The dots represent individual means.

We then investigated whether the theta timing between lip movements and sounds during movie encoding impacted information propagation between the sensory regions of the neocortex and the hippocampus. Although speech is not stationary, we aimed to test whether synchronous multisensory inputs during speech perception promoted network orchestration along the neocortical–subcortical pathway via theta oscillations. To do so, we estimated and compared the strength of theta phase coupling during synchronous or asynchronous movies, between the hippocampus and the auditory cortex, the visual cortex, or the cerebellum as a control region, all contained in the left hemisphere (Fig. 3*A*). Accordingly, we expected a greater theta phase coupling in the case of synchronous compared with asynchronous audiovisual speech, reflecting more optimal cross-region communication driven by the theta timing. Results revealed no significant effect of regions (*F*_(2,22)_ = 1.441; *p* = 0.248; *η²* = 0.061), synchrony (*F*_(1,22)_ = 0.05; *p* = 0.826; *η²* = 0.002), or interaction regions × synchrony (*F*_(2,22)_ = 0.430; *p* = 0.654; *η²* = 0.019), which in the present case did not support our hypothesis of theta-driven orchestration of brain networks during speech encoding (Fig. 3*B*).

### Audiovisual theta synchrony during encoding decreased the accuracy of speech replay from memory

**Figure 4.**
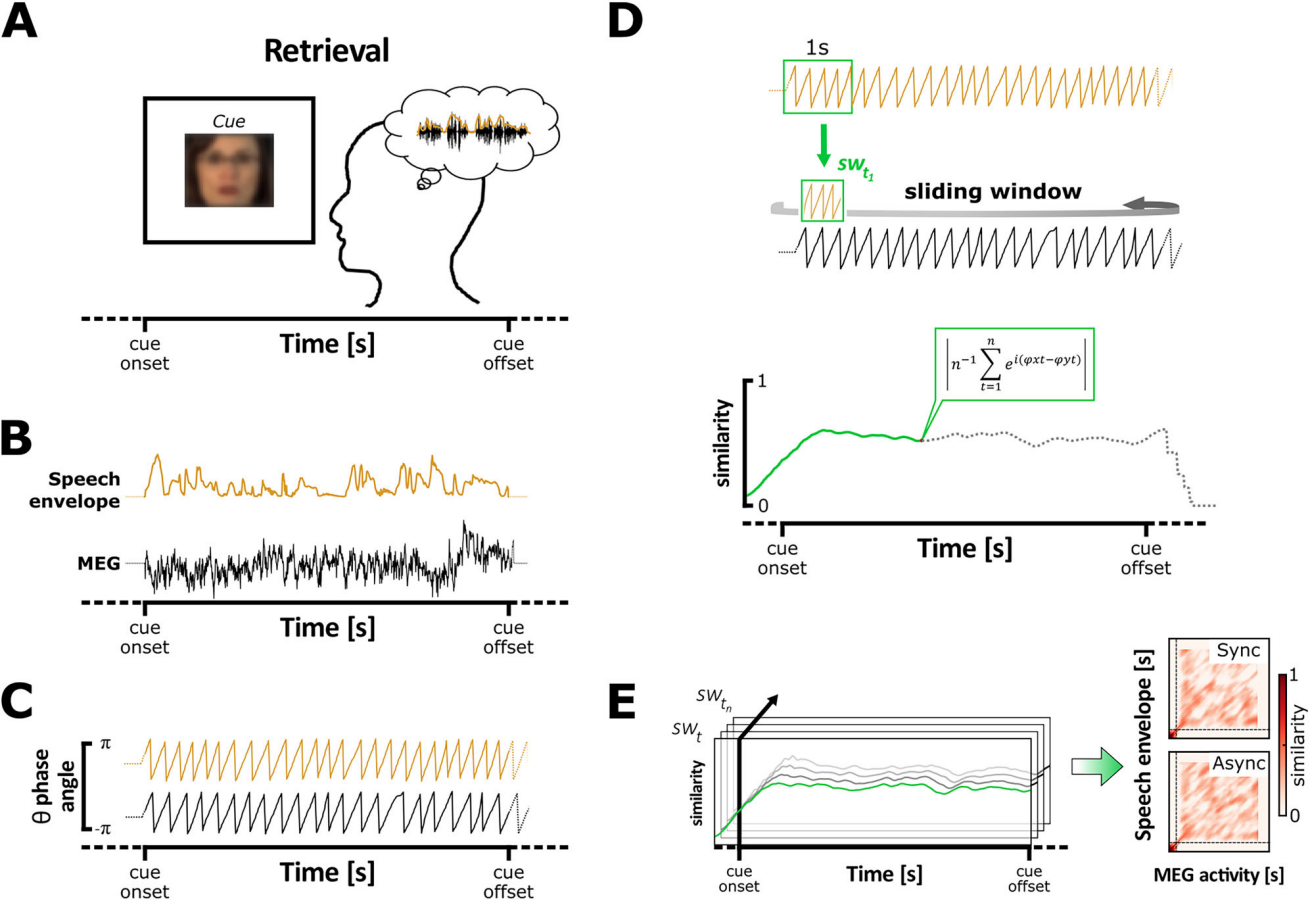
Measure of speech replay accuracy during retrieval. ***A***, During successful retrieval, participants recalled the auditory features associated with the speaker’s face, theoretically leading to mentally replaying “verbatim” the speech memorized during movie encoding. ***B***, In this case, the brain activity in the auditory cortex (MEG; black line) reinstates the theta oscillatory patterns conveyed by the auditory speech envelope of the movie (speech envelope; orange line). ***C***, The accuracy of speech reinstatement is measured with the level of phase similarity between the theta oscillation of the stimulus auditory envelope, and the neural theta oscillation produced replay of speech memory. To do that, the phase of the dominant theta frequency was extracted from the auditory envelope of the stimulus (orange line) and the MEG signal at the auditory sensors (black line). ***D***, The phase similarity was estimated between the first sliding window (sw) of 1 s containing the theta phase of the auditory envelope centered on its first time-point (sw*_t1_*) and every time-point of the MEG signal (top). The phase similarity was represented with a single value ranging between 0 and 1 at each time-point of the MEG signal (bottom). ***E***, This operation was repeated by shifting the sliding window to the next time-point (sw*_t2_*) of the auditory envelope signal and so on until its last time-point (sw*_tn_*), in order to compute the phase similarity between the two signal dimensions over time (left). Theta phase similarity was then averaged across windows, trials, and sensors of interest for statistical analyses (right). Adapted from Michelmann et al. (2016).

We then examined whether theta patterns from auditory cortices reflect the accuracy of replay of the speech envelope from memory, i.e., when participants recalled auditory information cued by the face of the speaker (see Fig. 4 for a description of the approach). We first needed to confirm that neural theta oscillations tracked the dominant activity conveyed in the auditory speech envelope during encoding, i.e., initial movie perception. We expected to find a theta “tracking” (i.e., alignment of theta phase with physical stimulus) in the primary auditory cortex (Heschl’s gyrus) during encoding.

**Figure 5.**
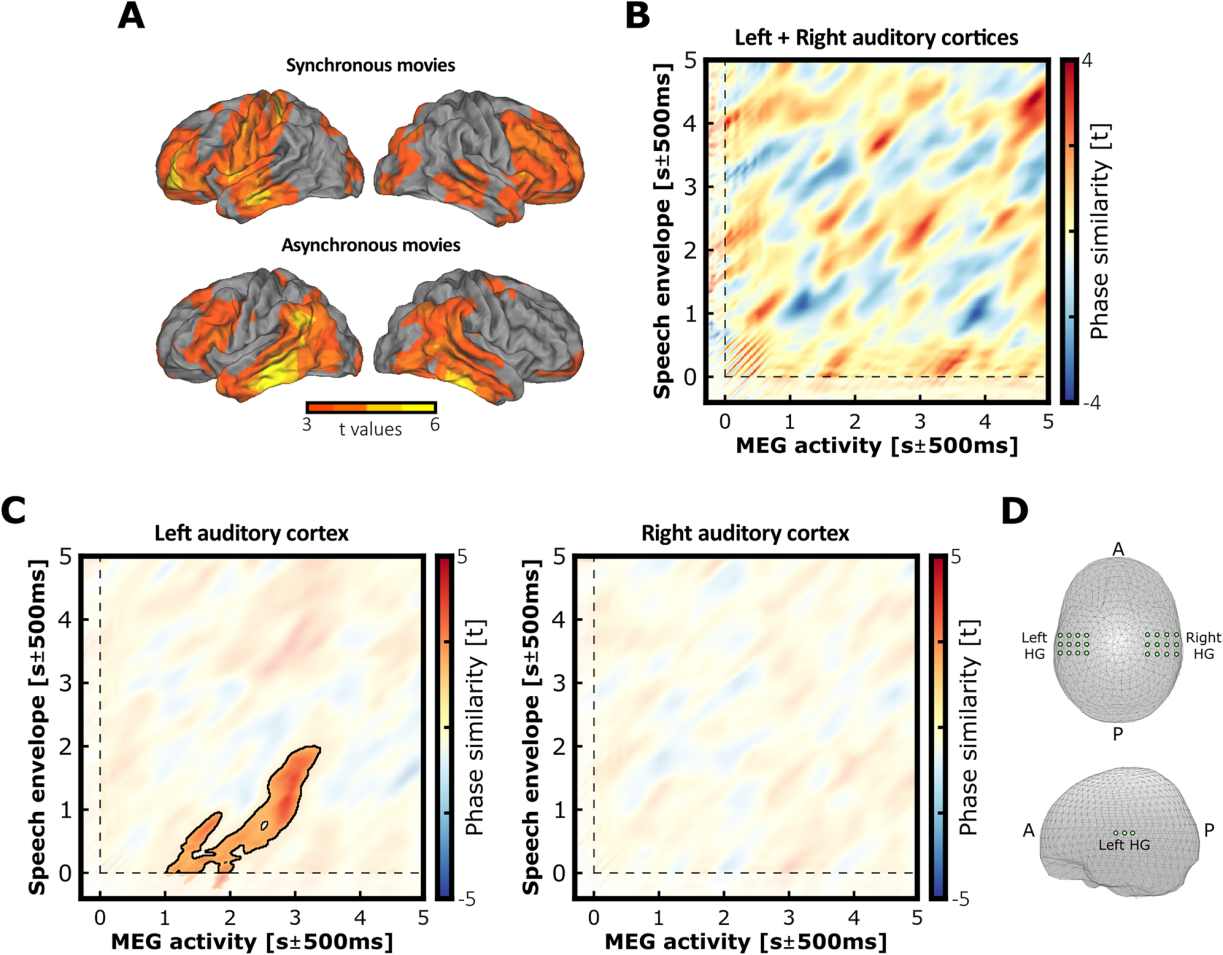
Theta oscillations track and reinstate the dominant activity carried in the auditory envelope. ***A***, Source localization of theta phase similarity between the brain oscillations and auditory speech envelope during synchronous or asynchronous movie encoding (significant *t* values). The tracking of the dominant theta oscillations carried by the auditory speech envelope was reflected across extended bilateral neocortical networks and similarly during synchronous and asynchronous movie, which suggests that audiovisual asynchrony did not impact auditory tracking per se. ***B***, Theta phase similarity between the theta oscillations from the left and right auditory cortices (*X*-axis) and the theta oscillations conveyed by auditory speech envelope (*Y*-axis) during successful retrieval (*t* values). The *X*-axis represents time for the MEG theta phase, and the *Y*-axis represents time for the speech envelope. ***C***, Theta phase similarity difference between the synchronous and asynchronous conditions in the left and right auditory cortices during successful retrieval. The accuracy of the reinstatement was modulated by the audiovisual asynchrony in the left but not the right auditory cortex. The time-points from the significant cluster are depicted in full color and outlined, while nonsignificant data are masked with transparency. ***D***, Virtual sources localized in the left and right auditory cortices used for the phase similarity analysis (respectively the left and right Heschl’s gyrus; LHG and RHG).

Figure 5*A* depicts the statistical difference of mean theta phase similarity (tps_Diff_) averaged across pairs of corresponding MEG and envelope signals from the same trials (matched) minus the mean theta phase similarity averaged across random pairing of signals coming from different trials (unmatched) during the encoding of synchronous or asynchronous movies (tps_Diff_ = tps_Matched_ − tps_Unmatched_). The results revealed a significant cluster when testing the phase similarity difference between matched and unmatched signals in both the synchronous (*p* < 0.0001; cluster size, 1.005 × 10^4^; mean *t* statistic within cluster, 3.133; Cohen’s *d*_z_ = 0.653) and asynchronous condition (*p* < 0.001; cluster size, 1.026 × 10^4^; mean *t* statistic within cluster, 3.212; Cohen’s *d*_z_ = 0.670). To address whether audiovisual synchrony significantly affected auditory envelope tracking during movie encoding, the difference of theta phase similarity difference between conditions (tps_Diff Sync_ − tps_Diff Async_) was computed at every virtual source for each participant. The difference of theta phase similarity difference for each participant was then entered into a two-tailed cluster–based permutation test as in the original analyses. Result revealed no significant cluster, suggesting that audiovisual asynchrony during movie encoding did not impact auditory signal tracking per se. These results also showed that during the encoding of audiovisual movies, neural theta oscillations tracked the dominant activity conveyed by the auditory speech signal across a broad neocortical network. Although not exclusive, the greatest theta tracking was situated bilaterally in the temporal regions including the primary auditory cortex, i.e., left and right Heschl’s gyrus, as well as pre- and postcentral areas. This supports the idea that theta asynchrony does not affect separate single signal processing (auditory and visual) but how they are integrated in neocortical regions.

Second, we aimed to compare the accuracy of replay of auditory speech envelopes between synchronous and asynchronous movies in auditory cortices. The rationale behind this analysis is that the auditory cortices track and encode theta oscillations carried by the auditory envelope during speech perception. Later, during retrieval, visually cueing an audiovisual speech memory should induce the reinstatement of brain activity representing the associated auditory information in the same auditory regions where it was previously encoded. This would be evidenced by the replay of identical theta oscillation patterns. If this is the case, audiovisual asynchrony during speech processing should disrupt the optimal encoding of theta oscillations carried by the auditory envelope, thereby reducing the fidelity of their reinstatement during retrieval compared with the synchronous condition. To this end, the theta phase similarity between every time-point of the MEG and corresponding speech envelope signals was computed in the left and right auditory cortices during the successful retrieval (Fig. 5*C*). Results revealed higher theta phase similarity for synchronous compared with asynchronous movies in the left auditory cortex (*p* = 0.023; cluster size, 1.22 × 10^4^; mean *t* statistic within cluster, 2.226; Cohen’s *d*_z_ = 0.464). This result confirmed that the accuracy of speech replay in the left auditory cortex was decreased when movies were encoded in the asynchronous as compared with the synchronous condition (Fig. 5*C*, **left**). In contrast, the cluster-based permutation test revealed no significant difference between the synchronous and asynchronous conditions in the right auditory cortex (Fig. 5*C*, **right**). For completeness, we tested whether the fidelity of speech envelope reinstatement in the auditory regions predicted memory during the retrieval. We expected to find a greater theta phase similarity for movies successfully retrieved (hit) as compared with forgotten (miss) movies. We applied the same analytic approach as above by comparing the difference of theta phase similarity between remembered and forgotten stimuli. Permutation tests revealed no significant cluster. However, the number of hit/miss trials was counterbalanced with a subsample corresponding to the smallest number of available miss trials for each participant, given the high correct response rates in the retrieval performances. This allowed few trials to compute theta phase similarity for hit and miss cases in each participant, which might have weakened statistical power in this contrast.

In a last analysis, we assessed whether theta oscillations from the auditory regions spontaneously replayed theta patterns encoded from auditory envelopes during movie presentation and independently from audiovisual synchrony. For each participant, we first computed the phase similarity between pairs of matching signals for synchronous and asynchronous trials together at the sources contained in the left and right auditory cortices (original data). Second, we computed the phase similarity between random pairs of unmatching signals for synchronous and asynchronous trials together. This step was repeated 100 times by generating a new set of random unmatching pairs for each iteration, and the resultant phase similarity was averaged across the 100 iterations (permuted data). The statistical difference of theta phase similarity between original and permuted data was then assessed with a one-tailed cluster–based permutation test across participants (Fig. 5*B*), which revealed no significant cluster. According to our hypothesis, misaligning visual streams negatively impacted auditory signal tracking during asynchronous movie encoding. If so, neural theta oscillations from the auditory cortex encoded and replayed theta oscillations conveyed in the auditory envelope less effectively than during synchronous encoding. It is possible that collapsing synchronous and asynchronous trials together decreased the resultant theta phase similarity during retrieval, by adding more inaccurate replays in the data (and potential not memory-driven), explaining the absence of significance for this last analysis.

Altogether, our results established that the auditory cortex reinstated theta phase patterns imposed by speech envelope differently depending on audiovisual synchrony in the previous movies. This suggests that the theta dynamics were encoded as relevant speech features contributing to memory replay.

## Discussion

In everyday life, we form an abundant amount of speech memories during casual conversations or when we see a movie. Replaying later what someone said and how they said it without much effort requires the brain to form an integrated representation of multisensory speech in memory. Theta oscillations in the neocortex and the hippocampus have shown to play an important role for episodic memory formation. Moreover, syllable information organizes speech on equivalent theta oscillations that align lip movements and auditory envelope. We go beyond previous work in showing that theta synchrony between speech and neuronal activation in neocortex and hippocampus reflects whether audiovisual speech was encoded in synchrony or not. Furthermore, we found that audiovisual asynchrony during encoding affects the accuracy at which speech stimuli are later replayed from memory.

Decreasing audiovisual theta phase alignment in the movies attenuated neural responses in right temporal regions including posterior middle temporal and superior temporal gyri, which are commonly associated with multisensory integration (Calvert et al., 2000; Callan et al., 2004; Macaluso et al., 2004; Meyer et al., 2004; Campbell, 2008; Nath and Beauchamp, 2012; Jansma et al., 2014). These areas often exhibit early superadditive activations within the first 160 ms in response to congruent audiovisual speech perception compared with unimodal speech (Gao et al., 2023; Salmi et al., 2017; Besle et al., 2008, 2009; Molholm et al., 2002). Further studies also reported that audiovisual integration takes place in both auditory and visual areas of the neocortex (Ghazanfar and Schroeder, 2006; Raij et al., 2010; Petro et al., 2017). For instance, Park et al. (2018) demonstrated that 3–7 Hz theta oscillations in the posterior superior temporal gyrus/sulcus (STG/S) encoded common features carried in auditory and visual inputs during audiovisual speech perception. Lip and sound onsets were misaligned by up to 125 ms in the stimuli, and the decrease of specific theta power found in the right temporal and visual regions suggests that theta asynchrony reduced audiovisual integration in both sensory and associative areas. Therefore, our results confirmed that the brain integrates lip-sound temporal mapping upon theta rhythm in the neocortex. Finally, the STG has been shown to encode acoustic–phonemic features during speech processing (Giraud and Poeppel, 2012; Yi et al., 2019).

Here, it is possible that desynchronizing lips and sounds affected acoustic–phonemic feature processing as well, because their dynamics depend on the syllable timing. This idea is corroborated by the theta activity tracking of the auditory envelope during movie encoding, which was found in the bilateral STG and was attenuated in the right hemisphere during the encoding of asynchronous movies. It is also possible that the audiovisual theta asynchrony imposed in the movies added a “visual noise” that shaped speech sound processing in the auditory associative STG/S regions (Holdgraf et al., 2016; Gwilliams et al., 2018).

Next, we showed that the natural theta timing in audiovisual speech determined the level of neural response synchrony down to the hippocampus as well. This was reflected by a decrease of power specifically in those theta frequencies where multisensory syllable information was asynchronous during movie encoding. Previous studies demonstrated that encoding of asynchronously flickering video–sound associations at theta predicted worse memories compared with synchronously flickering associations (Clouter et al., 2017; Wang et al., 2018). From the delay observed between the theta oscillation across cortical sensory areas, these studies hypothesized that the same theta timing was maintained downstream in the hippocampus to synchronize the responding neural activities and to promote audiovisual memory associations. Using MEG, the present study demonstrates hippocampal theta power modulations in response to the timing between multisensory speech features. This result was anticipated because during the encoding, the hippocampus receives multisensory information from the neocortex via the perforant pathway of the entorhinal region (Suzuki, 1996; Mayes et al., 2007; Moscovitch, 2008). In rodents and nonhuman primates, the auditory and visual sensory as well as associative cortical areas project to the parahippocampal and perirhinal cortices, which send information inputs to the entorhinal cortex (Witter and Amaral, 1991) which passes it on to the hippocampus. Cancelling theta synchrony in the frequency, organizing audiovisual speech movies is likely to affect multisensory integration normally taking place in neocortical associative areas, which would then pass on the misaligned information downstream to the hippocampus. It is worth noting that although MEG has traditionally been challenged with detecting activity originating in deep sources, recent studies have now established that MEG is indeed sensitive to signals from the hippocampus (Ruzich et al., 2019; Alberto et al., 2021; Joensen et al., 2023).

The results show that the neocortical theta phase tracked theta phase information carried in the speech envelope during movie encoding. Strikingly, this theta phase activity is later reinstated in the auditory cortices during subsequent retrieval. Interestingly, the audiovisual synchrony at encoding was associated with higher accuracy of speech replay at retrieval in the left auditory cortex. Using a similar analytic approach, a previous study showed the replay of auditory phase patterns at 8 Hz of music stimuli (Michelmann et al., 2016). In a verbal memory task, Yaffe et al. (2014) reported that theta oscillations from the bilateral temporal lobes mediated neural reinstatement associated with successful paired-word retrieval. However, words were encoded in isolation, and theta activity did not encode dynamic features of the event per se. Here, we extended these results with continuous speech stimuli by showing that the brain replays temporal patterns that organized speech information during encoding and that this replay depends on the temporal synchrony at initial encoding. Theta oscillations are thought to provide the optimal timing to promote LTP during the formation of new multisensory associations (Pavlides et al., 1988; Huerta and Lisman, 1995; Buzsaki, 2002; Hyman et al., 2003; Wang et al., 2023). The less accurate theta reinstatement observed when participants recalled asynchronous movies may reflect the weaker reactivation of memory traces as compared with synchronous movie memories. Nevertheless, recall performance in the subsequent memory test was not decreased by the audiovisual asynchrony during movie encoding. Therefore, our results have not confirmed our first prediction yet.

If audiovisual asynchrony reduced the accuracy of replay of speech stimuli, then why did it not affect memory performance? We see two possible answers. First, participants were able to distinguish between synchronous and asynchronous movies during encoding (Fig. 1*C*). Therefore, it is possible that asynchronous movies attracted listeners’ attention and artificially “boosted” audiovisual associations. Nevertheless, previous studies investigating the role of audiovisual synchrony with a theta rate of 4 Hz showed that the audiovisual asynchrony during encoding decreased subsequent memory performances, although participants could not discriminate between synchronous and asynchronous stimuli. This suggests that sensitivity to (a)synchrony did not predict memory performance (Clouter et al., 2017; Wang et al., 2018). Furthermore, splitting our data between discriminator and nondiscriminator populations based on their sensitivity index (*d*′) during movie encoding did not reveal any difference later during the memory task. Second, the memory task required participants to simply choose between two speech stimuli, which may not be sensitive to measure the accuracy of memory replay. For instance, a participant could reach very high levels of performance if they simply remember the rough content of what the speaker said (i.e., the speaker said something about “home-works”) as opposed to a fine-grained temporal representation of the speech content. Finally, although we employed a staircase procedure to adjust probe test difficulty to participants’ performance, it might be the case that this approach was simply not enough to limit the ceiling effect in our memory task. Future investigations should aim to improve the granularity of the memory task by testing participants on specific speech features which would be reflected in theta phase.

## Conclusion

We demonstrated that the encoding of speech movies in which the natural synchrony between speaker’s lip movements and sounds were preserved elicited stronger theta oscillations in the neocortex and the hippocampus. Furthermore, the replay of the temporal features encoded in the speech movies was mediated by the same theta oscillations. The accuracy of theta reinstatement during memory recollection was affected by the synchrony between lip movements and the auditory envelope during speech encoding. We conclude that neural theta oscillations play a pivotal role in both aspects of audiovisual speech memories, i.e., encoding and retrieval.

## Data Availability

Raw datasets, the codes for analyzing data, and reproducing related figures in the manuscript have been deposited at https://osf.io/4nh5w/ and will be made available after publication. Additional requests for information related to the present article will be directed to Emmanuel Biau (e.biau@liverpool.ac.uk).
